# Temperature Dependence of Anisotropy in Ti and Gd Doped NiMnGa-Based Multifunctional Ferromagnetic Shape Memory Alloys

**DOI:** 10.3390/ma13132906

**Published:** 2020-06-28

**Authors:** Amadeusz Łaszcz, Mariusz Hasiak, Jerzy Kaleta

**Affiliations:** Department of Mechanics, Materials and Biomedical Engineering, Wrocław University of Science and Technology, 25 Smoluchowskiego, 50-370 Wrocław, Poland; mariusz.hasiak@pwr.edu.pl (M.H.); jerzy.kaleta@pwr.edu.pl (J.K.)

**Keywords:** magnetic shape memory alloys, multifunctional alloys, Ni-Mn-Ga, magnetocrystalline anisotropy

## Abstract

The temperature dependence of magnetocrystalline anisotropy was investigated in detail for the polycrystalline Ni_50_Mn_25_Ga_25_, Ni_50_Mn_25_Ga_20_Ti_5_ and Ni_50_Mn_25_Ga_20_Gd_5_ ferromagnetic shape memory alloys in the temperature range of 50–400 K. The effective anisotropy constant was estimated from a series of high field magnetization curves based on the fitting procedure according to the law of approach to magnetic saturation. The low temperature martensitic phase was found to have a significantly higher anisotropy energy in comparison to a high temperature austenitic phase, which was observed through a sudden, distinct drop of anisotropy energy. The calculated values of the effective anisotropy constant were comparable to the results published by other authors. Moreover, the strong influence of chemical composition on the first-order phase transition and the second-order ferromagnetic to the paramagnetic transition was revealed. Finally, the strong coupling between the temperature dependence of the coercive field and the temperature dependence of magnetocrystalline anisotropy was also shown and discussed in the present study.

## 1. Introduction

Ferromagnetic shape memory alloys (FSMA) are recently one of the most extensively studied group of modern smart materials [1,2,3]. Among them, NiMnGa-based Heusler compounds stand out as the most complex alloys due to their unique magnetomechanical properties, such as magnetic field induced strains [4,5,6], pseudoelasticity/superelasticity [7,8,9], magnetoresistance [10,11] or magneto- and mechanocaloric effects [12,13,14,15,16]. All above mentioned properties are associated with the stress-, magnetic field- or temperature-driven first-order reversible phase transition, and undergo a change from a high symmetry austenitic phase to low symmetry martensitic phase [17]. Furthermore, a low temperature martensitic phase is characterized by strong magnetocrystalline anisotropy, which helps to induce and control the twin variant reorientation within the martensitic phase [18]. The strong correlation between the microstructure and the magnetic properties of the austenitic and martensitic phases leads to the abrupt drop of magnetization in the vicinity of the martensitic transformation. This significant difference in magnetization strongly influences the majority of magnetomechanical properties in NiMnGa-based materials. When considering potential multifunctional applications of FSMA, it is necessary to manipulate the magnetic behavior of the martensite and austenite phases. It is well documented that the magnetism of NiMnGa-based alloys varies significantly with composition in and near stoichiometric Ni_2_MnGa samples depending on the Ni [19,20,21], Mn [22,23] or Ga [24,25] concentration. However, it has been recently reported that an addition of the fourth alloying element may cause a significantly change in the magnetostructural properties of the existing phases [13,26,27]. The introduction of doping elements changes the stability of the austenite and martensite phases, which shifts the structural transformation temperatures and changes the temperature dependent magnetic behavior of doped NiMnGa-based materials. Moreover, alloying elements have different magnetic moments than primary Ni, Mn and Ga, which also influence the ferromagnetic properties of the material. Crucially, even a small amount of doping element can also notably shift the temperature of the martensitic transformation [28,29,30,31,32,33,34].

The present work aimed to investigate the influence of Ti and Gd substitution for Ga in polycrystalline Ni_50_Mn_25_Ga_20_X_5_ (X = Ti or Gd) FSMAs with a magnetic behavior of the martensite and austenite phases before, during, and after phase transitions. Due to the fact that magnetocrystalline anisotropy changes during structural transformation, we estimated the temperature dependence of anisotropy energy from the series of high field magnetization curves based on the law of approach to magnetic saturation [35].

## 2. Materials and Methods

The polycrystalline bulk samples with the nominal composition of Ni_50_Mn_25_Ga_25_, Ni_50_Mn_25_Ga_20_Ti_5_ and Ni_50_Mn_25_Ga_20_Gd_5_ (at.%) were produced from high purity elements via an arc-melting method under a protective argon atmosphere. The melting procedure was repeated several times to ensure a high homogeneity of the samples. Despite the fact that Mn is a highly volatile element, the weight loss after the melting procedure was less than 1% for each sample. The produced ingots were subsequently vacuum-sealed in a quartz ampule and annealed at 1173 K for 5 h, followed by water quenching. Our previous studies [30] showed that Gd-doped NiMnGa-based FSMA require a higher annealing temperature, so to ensure a good homogeneity in the Ni_50_Mn_25_Ga_20_Gd_5_ sample, the alloy was further annealed at 1430 K for 3 h and then quenched in water.

The nominal compositions of the Ni_50_Mn_25_Ga_25_, Ni_50_Mn_25_Ga_20_Ti_5_ and Ni_50_Mn_25_Ga_20_Gd_5_ alloys were confirmed with the help of a Scanning Electron Microscope JEOL JSM-5800LV (JEOL Ltd., Akishima, Tokyo, Japan) supported by an energy-dispersive detector (EDS). The crystal structure identification was done on the basis of powder X-ray diffraction measurements at room temperature performed in the X-ray Diffractometer Rigaku MiniFlex 600 (Rigaku, Tokyo, Japan)) with CuKα radiation, followed by subsequent Rietveld analysis. The magnetic properties characterization was carried out using the Vibrating Sample Magnetometer (VSM) module from the VersaLab System (Quantum Design, San Diego, CA, USA). In order to reduce the influence of the demagnetizing field, the thin, needle-like samples (with a length to diameter ratio of more than 10) were used for the magnetic measurements. The temperature dependence of magnetization under an external magnetic field of 250 mT was measured from 50 K up to 400 K using zero-field cooled (ZFC) and field cooled (FC) protocols with heating and cooling rates of 10 K/min. Then, the series of magnetic hysteresis loops was also recorded in the same temperature range (50–400 K) under an external magnetic field of up to 2 T.

## 3. Results and Discussion

### 3.1. Microstructure Analysis

**Figure 1:**
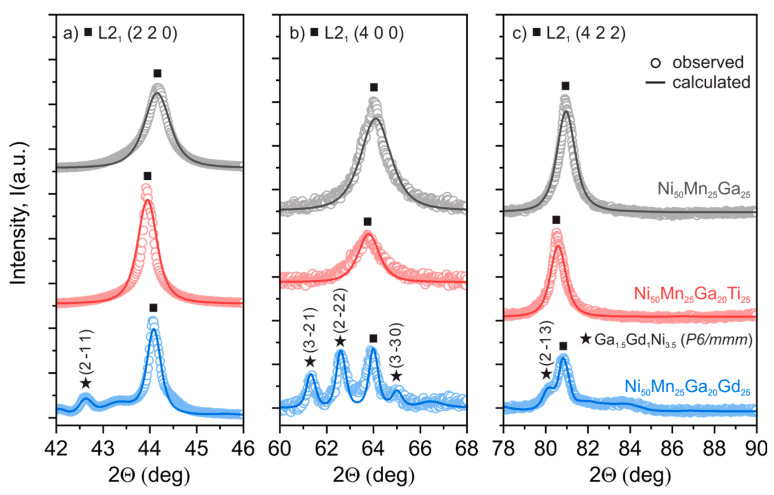
XRD patterns of the three peaks of the L2_1_ austenitic phase (**a**) (2 0 0), (**b**) (4 0 0) and (**c**) (4 2 2) obtained for the Ni_50_Mn_25_Ga_25_, Ni_50_Mn_25_Ga_20_Ti_5_ and Ni_50_Mn_25_Ga_20_Gd_5_ alloys. The dotted line represents the measured values and the solid line shows the calculated pattern obtained from the Rietveld analysis. The additional peaks observed only in the Ni_50_Mn_25_Ga_20_Gd_5_ alloy are related to the residual Gd-rich Ga_1.5_Gd_1_Ni_3.5_ phase (space group P6/mmm).

Figure 1 depicts the observed and calculated XRD patterns obtained at room temperature for the investigated Ni_50_Mn_25_Ga_25_, Ni_50_Mn_25_Ga_20_Ti_5_ and Ni_50_Mn_25_Ga_20_Gd_5_ alloys. In order to clearly show the slight deviation of the peak locations in each pattern, only three peaks with the highest intensity are presented in Figure 1. The presented reflections were assigned to a standard cubic L2_1_-type austenitic structure with an Fm3m space group (No. 225). In this case Ni, Mn and Ga occupied the 8*c* (0.25, 0.25, 0.25), 4*a* (0, 0, 0) and 4*b* (0.5, 0.5, 0.5) Wyckoff atomic positions, respectively. The lattice parameters calculated from the Rietveld analysis are as follows: *a* = 5.826 Å for Ni_50_Mn_25_Ga_25_, *a* = 5.848 Å for Ni_50_Mn_25_Ga_20_Ti_5_ and *a* = 5.832 Å for Ni_50_Mn_25_Ga_20_Gd_5_ alloys. It can be seen that an addition of doping elements (Ti or Gd) leads to the slight expansion of the austenitic unit cell. Moreover, in the case of the Ni_50_Mn_25_Ga_20_Gd_5_ sample, additional peaks were observed in the XRD pattern. These reflections were assigned to a residual hexagonal Ga_1.5_Gd_1_Ni_3.5_ phase (space group P6/mmm, No. 191) precipitated during the annealing process. The lattice parameters calculated for this phase are *a* = 4.980 Å, *c* = 4.107 Å and *γ* = 120°.

### 3.2. Temperature Dependence of Magnetization

**Figure 2:**
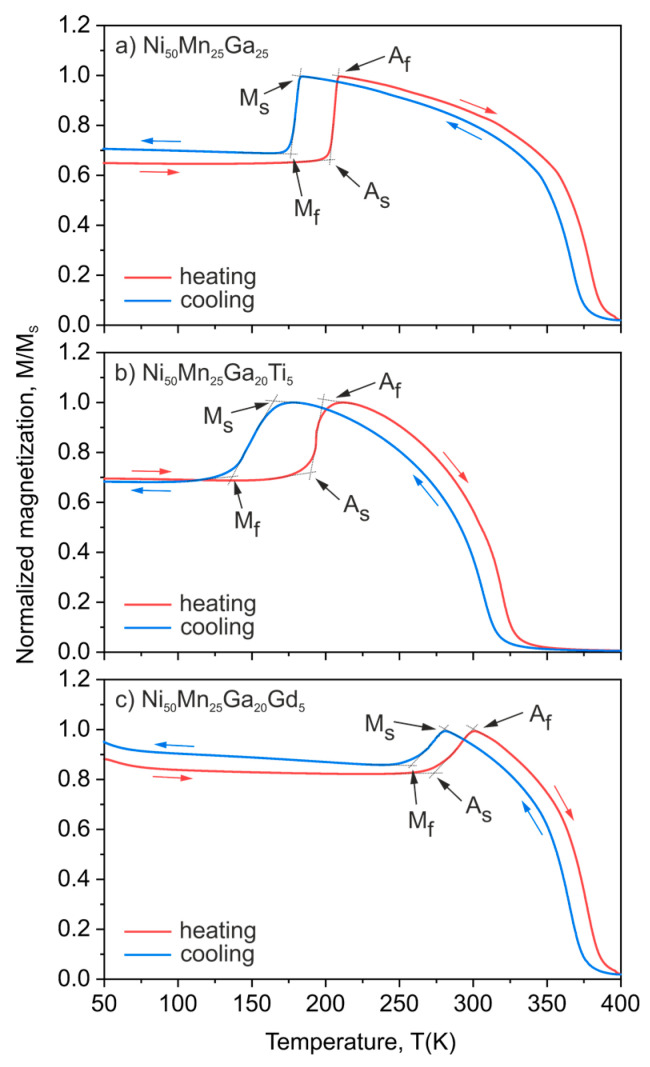
Temperature dependence of normalized magnetization for the (**a**) Ni_50_Mn_25_Ga_25_, (**b**) Ni_50_Mn_25_Ga_20_Ti_5_ and (**c**) Ni_50_Mn_25_Ga_20_Gd_5_ alloys measured under an external magnetic field of 250 mT. The arrows represent the austenitic transformation’s start/finish temperatures (*A_s_*/*A_f_*) and the martensitic transformation’s start/finish temperatures (*M_s_*/*M_f_*).

Figure 2 shows the temperature dependence of magnetization for the investigated Ni_50_Mn_25_Ga_25_, Ni_50_Mn_25_Ga_20_Ti_5_ and Ni_50_Mn_25_Ga_20_Gd_5_ alloys. A notable change of magnetization occurred in the vicinity of the phase transition in every investigated sample, both during heating and cooling procedures. The substantial hysteresis between the temperature of the martensitic transformation (during cooling) and the austenitic transformation (during heating) was very characteristic for the first-order temperature-driven transformation, especially in polycrystalline materials where the transition takes place in every single grain. Moreover, the martensitic transformation temperature was strongly dependent from the Gibbs free energy difference between the austenite and martensite phases and the addition of alloying elements may have significantly changed these energies [36,37,38]. In order to compare the transformation temperatures between the studied alloys, the average value of all temperatures (*M_s_*, *M_f_*, *A_s_*, *A_f_*) was calculated as:

(1) $$T_{M}=\frac{1}{2}\left(\frac{M_{s}+M_{f}}{2}+\frac{A_{s}+A_{f}}{2}\right),$$

where *T_M_* is structural transformation temperature, *M_s_* and *M_f_* are martensitic transformation start and finish temperature, and *A_s_* and *A_f_* are austenitic transformation start and finish temperature.

As was expected, both Ti and Gd substitutions for Ga notably shifted the phase transformation temperatures (*T_M_*). Ti slightly decreased the *T_M_* from 193 K to 173 K for Ni_50_Mn_25_Ga_25_ and Ni_50_Mn_25_Ga_20_Ti_5_ alloys respectively. On the other hand, the same atomic amount of Gd substantially increased the *T_M_* to 279 K for the Ni_50_Mn_25_Ga_20_Gd_5_ alloy. These results suggest that the addition of Ti decreases the overall free energy difference between the martensite and austenite phases, which subsequently results in a decrease of the martensitic transformation temperature. In a similar manner, the addition of Gd notably increases the free energy difference between the austenite and martensite phases, which leads to a considerable increase in the martensitic transition temperature. Moreover, in the case of the Gd-doped sample, the temperature of the phase transition was very close to the room temperature; this may be an advantage in potential future applications of this material.

Furthermore, the influence of Ti and Gd on magnetic transformation is significantly different than it is on structural transitions. The Curie temperature (calculated as an average value of the peak location on the first derivative’s *dM*/*dT* curves established during the heating and cooling protocol) dropped substantially in the case of the Ti-doped sample (*T_C_* = 373 K and 312 K for the Ni_50_Mn_25_Ga_25_ and Ni_50_Mn_25_Ga_20_Ti_5_ alloys, respectively). When it comes to the Ni_50_Mn_25_Ga_20_Gd_5_ alloy, the Curie temperature remained almost unchanged (*T_C_* = 374 K).

### 3.3. Law of Approach to Magnetic Saturation

The well-known law of approach (LoA) to magnetic saturation was used to analyze the magnetic behavior of all of the studied alloys and to estimate the temperature dependence of their effective magnetocrystalline anisotropy [35]. However, in order to properly use the LoA model, the authors made some important assumptions. First of all, only the high field magnetization curves (*H* ≫ *Hc*) were taken into account as initial curves. Next, after the first calculations were made considering the fitting procedure accuracy, the high field magnetization curves were limited to the last 5% of magnetization (*M* > 0.95*M_s_*). Further, in this applied range of magnetization, recorded hysteresis loops were closed so that all hysteretic processes could be neglected, since further increase of magnetization was mainly induced by rotational processes of magnetic domains. Properly selected magnetization curves were then analyzed in terms of the law of approach to magnetic saturation [35]:

(2) $$M=M_{s}\left(1-\frac{a}{H}-\frac{b}{H^{2}}\right)+\chi H,$$

where *M_s_* is saturation magnetization, coefficient *a* is related to the to the structural inhomogeneity of the material, non-magnetic inclusions or internal microstresses, and coefficient *b* is directly connected with the existence of magnetocrystalline anisotropy. The last term of the equation *χH* describes the high field spontaneous magnetization. Considering the fact that coefficient *a* plays a significant role only in the lower fields [35,39] and its contribution at high magnetic fields could be neglected, Equation (2) may be simplified to:

(3) $$M=M_{s}\left(1-\frac{b}{H^{2}}\right)+\chi H,$$

**Figure 3:**
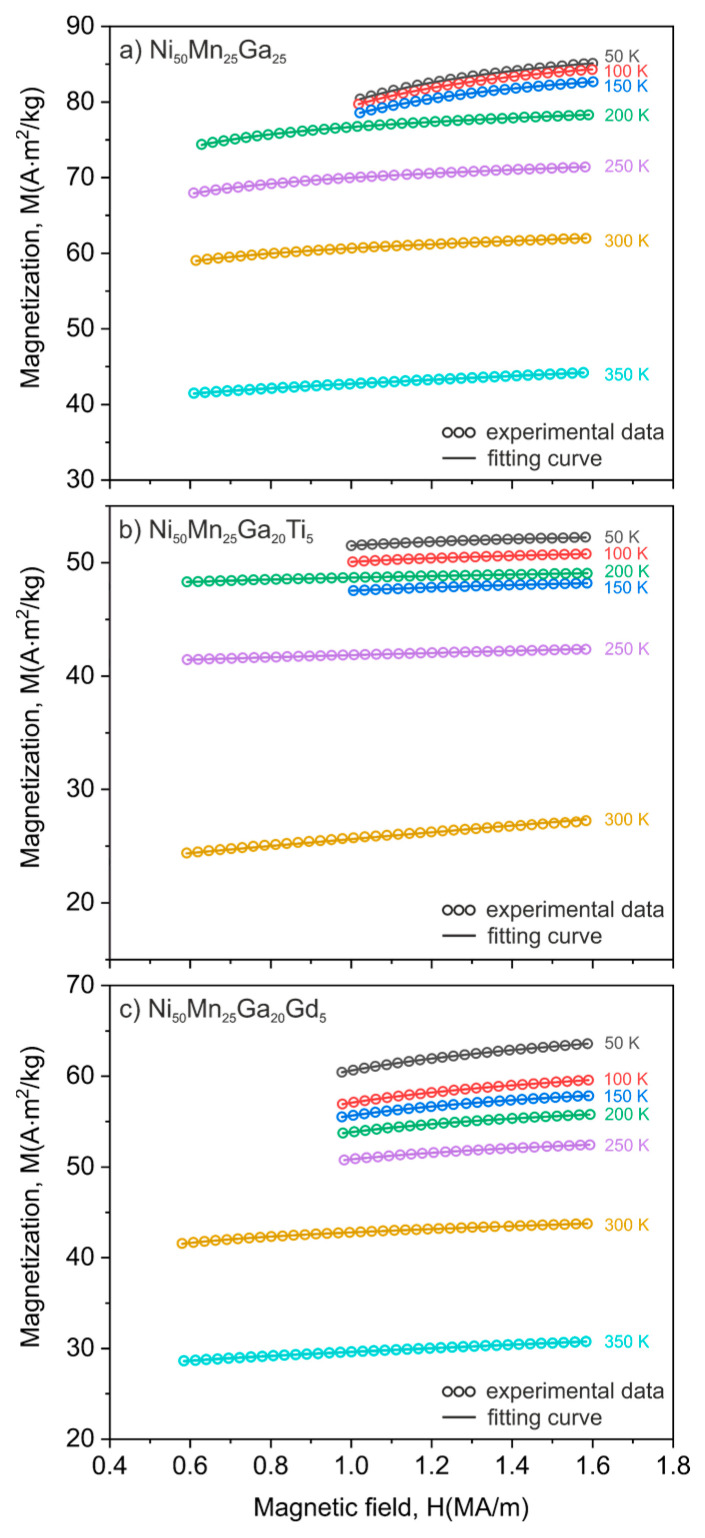
High field parts of magnetization curves (*M* > 0.95*M_s_*) for the (**a**) Ni_50_Mn_25_Ga_25_, (**b**) Ni_50_Mn_25_Ga_20_Ti_5_ and (**c**) Ni_50_Mn_25_Ga_20_Gd_5_ alloys measured from 50 K up to the Curie temperature. Open circles represent recorded data and solid lines show the best fitting to the law of approach to magnetic saturation (see Equation (3)).

Equation (3) was used to fit to the selected part of the magnetization curves, which is depicted in Figure 3. The open circles show the measured data and the solid lines represent the best fittings according to Equation (3). The significant difference between the magnetization behavior of the low temperature martensite phase and the high temperature austenite phase is observable for the all studied alloys. As expected, even though martensite is characterized by a higher magnetization at high values of an applied magnetic field, it saturates much slower than notably softer austenite. This reflects the fact that high magnetocrystalline anisotropy of the martensite phase decreases the initial permeability of the material at low magnetic fields. Coefficient *b* calculated from Equation (3) was used to estimate the effective magnetic anisotropy constant (*Keff*) from the following relation:

(4) $$b=c{\left(\frac{K_{eff}}{\mu_{0}M_{s}}\right)}^{2},$$

where *c* is a constant dependent from the crystal structure of the material and equals *c* = 8/105 for cubic anisotropy [35]. Thus, *K_eff_* can be finally estimated by utilizing the following formula:

(5) $$K_{eff}=\mu_{0}M_{s}\sqrt{\frac{105c}{8}},$$

**Figure 4:**
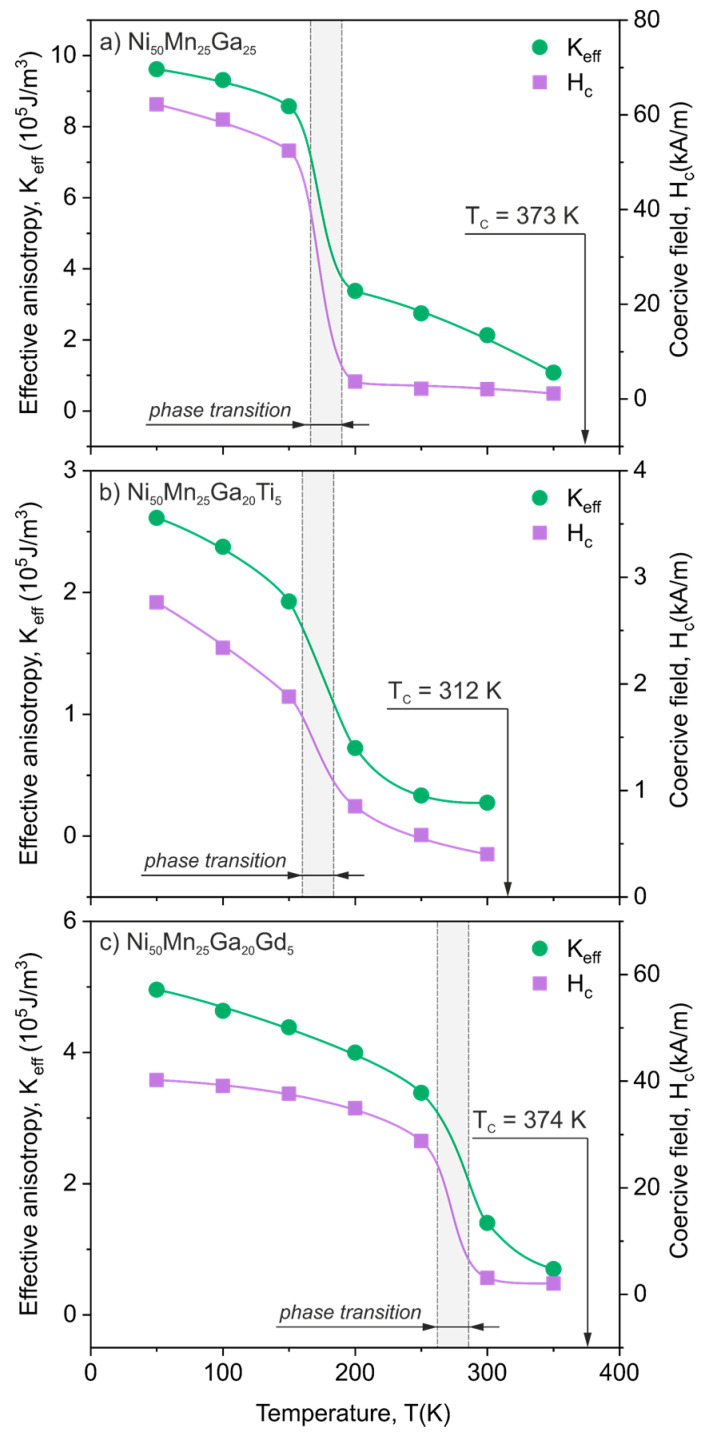
The temperature dependence of effective anisotropy constant *K_eff_* (circles) estimated from the law of approach to magnetic saturation (see Equation (5)) and temperature dependence of coercive field (squares) for the (**a**) Ni_50_Mn_25_Ga_25_, (**b**) Ni_50_Mn_25_Ga_20_Ti_5_ and (**c**) Ni_50_Mn_25_Ga_20_Gd_5_ alloys.

Figure 4 presents the calculated effective magnetic anisotropy constant for all three Ni_50_Mn_25_Ga_25_, Ni_50_Mn_25_Ga_20_Ti_5_ and Ni_50_Mn_25_Ga_20_Gd_5_ alloys. A significant drop of anisotropy (about 60%) in the vicinity of the phase transition was evident for every studied material. It was also clearly seen that the anisotropy energy decreased with the increase of temperature in both low temperature martensitic phases and high temperature austenitic phases (up to the Curie temperature where the material became paramagnetic). Moreover, the obtained values of anisotropy constant for the martensitic phase were the following: 9.6–8.5 × 10^5^ J/m^3^ for Ni_50_Mn_25_Ga_25_, 2.6–1.9 × 10^5^ J/m^3^ for Ni_50_Mn_25_Ga_20_Ti_5_ and 5.0–3.4 × 10^5^ J/m^3^ for Ni_50_Mn_25_Ga_20_Gd_5_ alloys; for the austenitic phase: 3.4–1.1 × 10^5^ J/m^3^ for Ni_50_Mn_25_Ga_25_, 0.7–0.3 × 10^5^ J/m^3^ for Ni_50_Mn_25_Ga_20_Ti_5_ and 1.4–0.7 × 10^5^ J/m^3^ for Ni_50_Mn_25_Ga_20_Gd_5_ alloys are in good agreement with the results reported for other NiMnGa-based materials [40,41,42,43]. The relatively high values of magnetocrystalline anisotropy in the martensitic phase were essential for magnetic field induced strains. The obtained results suggested that the additions of Ti and Gd to NiMnGa-based compositions significantly decreased the effective anisotropy constant. This was particularly apparent in the case of the Ni_50_Mn_25_Ga_20_Ti_5_ alloy in which *K_eff_* decreased about three times in comparison to the undoped Ni_50_Mn_25_Ga_25_ sample.

Figure 4 also depicts the temperature dependence of a coercive field obtained upon heating for Ni_50_Mn_25_Ga_25_, Ni_50_Mn_25_Ga_20_Ti_5_ and Ni_50_Mn_25_Ga_20_Gd_5_ alloys. In this case, the structural martensitic transition was also visible as a distinct drop in coercivity, which further showed the evident differences between the magnetic softness of the austenitic and martensitic phases. What is more in general, the coercive field was strongly coupled with the magnetic anisotropy of the material. This phenomenon is clearly observable in Figure 4, as the temperature dependence of the coercive field followed the same trend as the temperature dependence of effective anisotropy.

**Figure 5:**
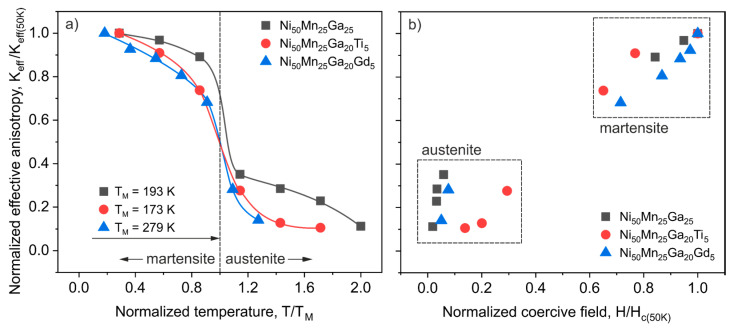
The normalized effective anisotropy constant (*K_eff_*/*K_eff(50K)_*) (**a**) as a function of normalized temperature (*T*/*T_M_*); the dashed line at *T*/*T_M_* = 1 shows the structural transformation, (**b**) as a function of the normalized coercive field (*H*/*H_c(50K)_*) for the Ni_50_Mn_25_Ga_25_, Ni_50_Mn_25_Ga_20_Ti_5_ and Ni_50_Mn_25_Ga_20_Gd_5_ alloys.

Another comparison of the temperature dependence of anisotropy is presented in Figure 5a, where an effective anisotropy constant was normalized by dividing it by a maximum value of *K_eff_* measured at 50 K; alternatively, the temperature axis was normalized by using the phase transformation temperature of each alloy. Thus, the dashed line at *T*/*T_M_* = 1 on Figure 5a corresponds to the structural transformation. The presented normalization procedure revealed that despite the significant differences in *K_eff_* and *T_M_*, the overall temperature dependence of effective anisotropy behaved very similarly in all studied materials and was strongly connected with the structural phase transition. Moreover, Figure 5b compares the normalized *K_eff_* with the normalized coercive field (*H*/*H_c(50K)_*). It was previously shown in Figure 5 that *K_eff_* is significantly related to the coercivity; this is even more evident now, in Figure 5b. The two characteristic regions for magnetically softer austenite and significantly harder martensite were easily distinguishable for all studied alloys.

## 4. Conclusions

The influence of elemental doping with Ti and Gd on the temperature dependence of magnetic behavior in the Ni_50_Mn_25_Ga_20-x_Z_x_ (x = 0 or 5, Z = Gd, Ti) ferromagnetic shape memory alloys were studied in detail. The XRD analysis confirmed the cubic L2_1_ austenitic structure in all investigated materials and revealed that an addition of Ti or Gd elongates the lattice parameters of the austenite cell. Thermomagnetic measurements showed the strong influence of chemical composition on both martensitic and magnetic transformations. Following this fact, Ti addition to the Ni-Mn-Ga alloy significantly reduces the martensitic transformation temperature and Curie temperature in comparison to the Ni_50_Mn_25_Ga_25_ precursor. In the Gd-doped sample, the temperature of magnetic transition remained almost the same as in the Ni_50_Mn_25_Ga_25_ alloy, whereas the temperature of phase transition increased significantly, and came close to room temperature. Comprehensive studies in high magnetic fields based on the law of approach to magnetic saturation theory revealed the close correlation between magnetic and structural behavior in investigated samples. The abrupt drop of magnetocrystalline anisotropy at phase transformation temperatures and significant differences of anisotropies between martensitic and austenitic phases confirmed the completely different magnetic nature of the two existing phases in NiMnGa-based FSMA. What is more, the strong connection between the temperature dependence of the coercive field and the temperature dependence of magnetocrystalline anisotropy was also emphasized in this study.
